# Adaptive immunotherapeutic paradigms in diffuse midline glioma: integrating epigenetic reprogramming, neuron–glioma interactions, and tumor microenvironment modulation

**DOI:** 10.1007/s11060-025-05347-9

**Published:** 2025-12-29

**Authors:** Justin Liu, Joseph H. Ha, Matthew Abikenari, Matthew Adam Sjoholm, Shreyas Annagiri, Karthik Ravi, Brandon H. Bergsneider, Rohit Verma, Debebe Theodros, Ravi Medikonda, Gordon Li, Laura M. Prolo, Michelle Monje, Michael Lim

**Affiliations:** 1 Department of Neurosurgery, Stanford University School of Medicine, Palo Alto, Stanford, CA 94304 USA; 2 Department of Neurology and Neurological Sciences, Stanford University, Stanford, California 94305 USA

**Keywords:** Immunotherapy, Neurosurgery, Oncology, Neuroscience, Diffuse midline gliomas, Diffuse intrinsic pontine glioma, CAR T cell, Vaccine, Radioimmunotherapy

## Abstract

**Background:**

Diffuse midline gliomas, including diffuse intrinsic pontine gliomas, represent one of the most aggressive pediatric malignancies in the central nervous system with a uniformly poor prognosis. They can be consistently identified by mutations in histone H3 K27M, which are associated with aggressive tumor biology, marked resistance to therapies, and abysmal survival. The current review critically assesses the existing application of immunotherapeutic modalities in DMGs, emphasizing biological hurdles in efficacy, translation methodologies, and prospects in attaining sustained responses.

**Methods:**

We examined preclinical and early clinical studies in DMGs for immune therapies such as peptide vaccines against H3K27M antigens, chimeric antigen receptor T-cell therapies, immune checkpoint modulation, and radioimmunotherapy. Current developments in the interface of cancer neuroscience and tumor interaction with neurons were incorporated in a manner relevant to immune suppression in the microenvironment of DMG. Although these tumors have traditionally shown poor immune reactivity because of low tumor mutational burden, immune-privileged sites, and a strongly suppressive tumor microenvironment, a variety of different immune therapeutic approaches have shown promising early efficacy. Of particular interest are neoantigen-targeted vaccines and CAR T-cell therapy using surface antigens. Preliminary findings suggest an important role for neuron–glioma synaptic and paracrine signaling in mediating tumor progression and immune evasion.

**Conclusions:**

Immunotherapy for DMGs is moving from a conceptual state to a translational reality. A better understanding of the realm of tumor immune–neural crosstalk, combination therapies, and immune biology in pediatric patients will be critical in addressing resistance and providing durable control for these aggressive malignancies.

## Introduction

The 5th edition of the WHO Classification of Tumours defines diffuse midline glioma (DMG), H3 K27-altered, as an infiltrative midline tumor showing loss of H3 p.K28me3 (K27me3). DMGs must also exhibit an H3 p.K28M (K27M) mutation, a p.K28I (K27I) mutation, EZHIP overexpression, EGFR mutation, or a methylation profile consistent with one of these alterations. These tumors most commonly arise in the brainstem and are termed diffuse intrinsic pontine gliomas (DIPGs) when located in the pons. They may also occur in the thalamus or spinal cord, and less frequently in the pineal gland, hypothalamus, or cerebellum [1]. Despite decades of research, these DMG remains among the most lethal of pediatric brain tumors, with a median survival of less than one year despite decades of research[]. Despite decades of research, these DMG remains among the most lethal of pediatric brain tumors, with a median survival of less than one year despite decades of research [2]. The clinical course of DMGs are uniformly aggressive: symptoms often develop rapidly, and patients typically decline within months of diagnosis. The rapid neurological deterioration, absence of effective therapeutic interventions, and predictably fatal trajectory of the disease has seen little-to-no progress over the past several decades. The unique biology of DMG includes its location, infiltrative nature, and molecular profile, which has historically limited therapeutic advances. Surgical resection is not feasible due to anatomical constraints, and conventional chemotherapies have offered no benefit due largely in part to the intact blood brain barrier [3].

To-date, radiation therapy remains the only standard-of-care treatment, providing transient symptom relief and extension of overall survival by about 3 months [4]. As such, there is an urgent need to develop novel therapeutic strategies for DMG. Immunotherapy, such as anti-PD-1/PD-L1 and anti-CTLA4 checkpoint blockade, has revolutionized the treatment landscape for hematological malignancies and several adult solid cancers, including melanoma and non-small cell lung cancer, and is being increasingly explored in central nervous system (CNS) tumors. Pediatric brain tumors, however, present unique challenges due to their low mutational burden, the suppressive immune microenvironment found within the tumor milieu, and developmental context [5].

This review focuses on emerging immunotherapeutic strategies for DMG, including immune checkpoint inhibitors, tumor vaccines, cellular therapies, and strategies to modulate the tumor microenvironment. Particular attention is given to both preclinical and early-phase clinical efforts that aim to overcome the resistance of DMG to immunotherapies and unlock durable antitumor responses. We concentrate on the disease background and subtypes of DMG, the landscape of tumor mutational burden and neoantigenicity, neuron–glioma Interactions, epigenetic reprogramming, and the immunologic barriers intrinsic to this disease, culminating in a critical appraisal of contemporary immunotherapeutic strategies, including immune checkpoint blockade and cellular therapies.

## DMG disease background & subtypes

The 2016 and 2021 WHO classifications of CNS tumors define diffuse midline glioma as a high-grade glioma characterized by diffuse growth and the presence of a K27M mutation in histone H3 genes, namely H3F3A or HIST1H3B/C [2, 6]. The mutation induces a global loss of H3K27 trimethylation (H3K27me3) through inhibition of the polycomb repressive complex 2 (PRC2), reprogramming the epigenetic landscape of affected cells [7, 8]. Although the term “DIPG” historically referred to tumors centered in the pons, DMG now encompasses histologically similar tumors arising in other midline structures, including the thalamus, cerebellum, and spinal cord [9]. This broader classification reflects shared molecular drivers and biological behavior, regardless of exact anatomical site, and reinforces the importance of molecular diagnostics in guiding classification and treatment.

DMG is predominantly a pediatric disease, typically affecting children between 5 and 10 years of age but can occur at any age, including adulthood. The clinical course is aggressive, with symptoms arising from brainstem involvement, such as cranial nerve deficits and long tract signs. Without treatment, median survival is approximately 3–6 months; with radiation, median overall survival extends to 9–12 months [4, 10]. Recent real-world data from an international, academia-driven compassionate-use cohort of ONC201 (dordaviprone)–containing regimens report improved overall survival of roughly 15 months for brainstem DMG and longer for thalamic DMG, though adult over-representation warrants caution in extrapolating to purely pediatric populations [11]. While the majority of DMGs harbor the H3.3 K27M mutation, alternative variants include H3.1 K27M and, more rarely, H3-wildtype tumors with epigenetic or genomic features that mimic DMG biology [12].

Comparative studies have shown that H3-wildtype midline gliomas are often biologically distinct, with more heterogeneous driver mutations (e.g., TP53, ACVR1, PPM1D) and variable clinical outcomes [13, 14]. Furthermore, H3.1 K27M tumors, commonly arising in younger patients, may portend slightly improved prognosis compared to their H3.3-mutant counterparts [15]. Notably, H3.1-mutant tumors tend to localize more frequently in the pons, while thalamic DMGs are more often H3.3-mutant, adding another layer of complexity to subtype classification [13–17]. Thus, stratification by H3 status and anatomical location is crucial in immunotherapy development, given differential expression of tumor-associated antigens and immune infiltration profiles across subtypes [18].

## Materials and methods

**Fig. 1:**
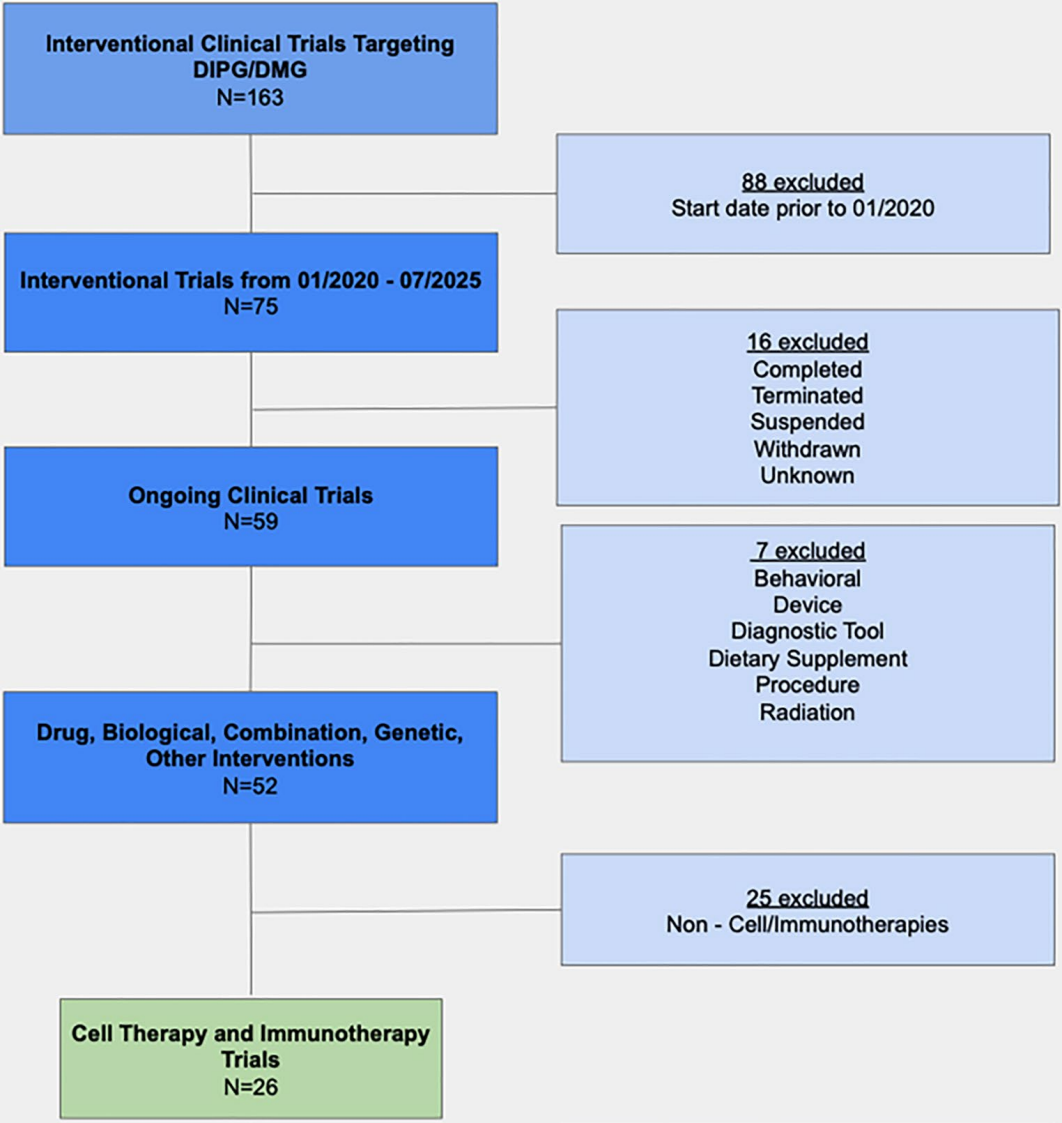
Systematic filtering and selection of DMG trials

A systematic search of the ClinicalTrials.gov database was performed to identify clinical trials investigating DMG cell-based or immunotherapy strategies, as summarized in Table 1. The search focused on studies registered between January 1, 2020, and June 2025. A total of 163 trials related to DMG were initially identified. To focus on recent and emerging treatment approaches, 88 studies with start dates prior to January 1, 2020, were excluded. Of the remaining 75 trials, those marked as completed, terminated, suspended, withdrawn, or listed with unknown status (*n* = 16) were removed, narrowing the pool to 59 actively ongoing studies. These were further screened based on intervention type, retaining only those involving biological agents, genetic therapies, pharmaceutical drugs, combination products, or other relevant treatment strategies (*n* = 52). Studies involving behavioral approaches, devices, diagnostic tools, dietary supplements, procedural methods, or radiation were excluded (*n* = 7). An additional 25 trials that did not involve cell-based or immunotherapy strategies were also removed. Ultimately, 26 clinical trials investigating novel immunotherapies for DMG were selected for in-depth review. Data from these studies were systematically organized and analyzed, focusing on trial phase, therapeutic type, specific interventions, patient demographics, endpoints, and projected timelines, offering a comprehensive overview of the current landscape in immunotherapy treatment development for DMG (Fig. 1, Table 2).

**Table 1:** Summary of ongoing immunotherapy clinical trials in DMG

NCT Number	Phases	Start Date	Primary Completion Date	Study Title	Study Status	Conditions	Age	Interventions	Study Description and Notes
NCT04196413	1	2020–06–04	2028–07–31	GD2 CAR T Cells in Diffuse Intrinsic Pontine Gliomas(DIPG) & Spinal Diffuse Midline Glioma(DMG)	Recruiting	Glioma of Spinal Cord, Glioma of Brainstem	Both Child (DIPG) and Adult CNS Tumor Patients	GD2 CAR T cells	The primary purpose of this study is to test whether GD2-CAR T cells can be successfully made from immune cells collected from children and young adults with H3K27M-mutant diffuse intrinsic pontine glioma (DIPG) or spinal H3K27M-mutant diffuse midline glioma (DMG).
NCT06305910	1	2024–03–15	2025–09–15	CD200AR-L and Allogeneic Tumor Lysate Vaccine Immunotherapy for Recurrent HGG and Newly Diagnosed DMG/DIPG in Children and Young Adults	Recruiting	Diffuse Midline Glioma, H3 K27M-Mutant, Recurrent High Grade Glioma	Both Child (DIPG) and Adult CNS Tumor Patients	CD200AR-L	This is a single center Phase I study of a new adjuvant CD200 activation receptor ligand, CD200AR-L, in combination with imiquimod and GBM6-AD vaccine to treat malignant glioma in children and young adults.
NCT04943848	1	2022–01–10	2025–06–01	rHSC-DIPGVax Plus Checkpoint Blockade for the Treatment of Newly Diagnosed DIPG and DMG	Recruiting	Diffuse Intrinsic Pontine Glioma, Diffuse Midline Glioma, H3 K27M-Mutant	Both Child (DIPG) and Adult CNS Tumor Patients	rHSC-DIPGVax	This is a phase I, open label, plus expansion clinical trial evaluating the safety and tolerability of rHSC-DIPGVax in combination with BALSTILIMAB and ZALIFRELIMAB.
NCT05768880	1	2023–05–05	2028–01–15	Study of B7–H3, EGFR806, HER2, And IL13-Zetakine (Quad) CAR T Cell Locoregional Immunotherapy For Pediatric Diffuse Intrinsic Pontine Glioma, Diffuse Midline Glioma, And Recurrent Or Refractory Central Nervous System Tumors	Recruiting	Diffuse Intrinsic Pontine Glioma, Diffuse Midline Glioma, Recurrent CNS Tumor	Both Child (DIPG) and Adult CNS Tumor Patients	SC-CAR4BRAIN	This is a Phase 1 study of central nervous system (CNS) locoregional adoptive therapy with SC-CAR4BRAIN, an autologous CD4+ and CD8+ T cells lentivirally transduced to express to express combinations of B7–H3, EGFR806, HER2, and IL13-zetakine chimeric antigen receptors (CAR).
NCT05298995	1	2023–11–09	2027–11–01	GD2-CAR T Cells for Pediatric Brain Tumours	Recruiting	Pediatric Brain Tumors	Both Child (DIPG) and Adult CNS Tumor Patients	GD2–CART01 (iC9–GD2-CAR T-cells)	The purpose of this study is to test the safety and efficacy of iC9–GD2-CAR T-cells, a third generation (4.1BB-CD28) CAR T cell treatment targeting GD2 in pediatric or young adult patients affected by relapsed/refractory malignant central nervous system (CNS) tumors.
NCT04911621	1,2	2021–09–10	2027–06–01	Adjuvant Dendritic Cell Immunotherapy for Pediatric Patients With High-grade Glioma or Diffuse Intrinsic Pontine Glioma	Active, not recruiting	DIPG	Child	Dendritic cell vaccination + temozolomide based chemoradiation Dendritic cell vaccination ± conventional next-line treatment	The general objective of this phase I/II clinical study is (1) to demonstrate that WT1-targeted DC vaccine production and administration in pediatric patients with HGG and DIPG is feasible and safe, (2) to study vaccine-induced immune responses, (3) to document patients’ quality of life and clinical outcome.
NCT06639607	1,2	2025–07–01	2030–09–30	PEP-CMV + Nivolumab for Newly Diagnosed Diffuse Midline Glioma/High-grade Glioma and Recurrent Diffuse Midline Glioma/High-grade Glioma, Medulloblastoma, and Ependymoma	Not yet recruiting	Diffuse Midline Glioma, Diffuse Midline High-grade Glioma, Medulloblastoma, Ependymoma	Both Child (DIPG) and Adult CNS Tumor Patients	PEP-CMV vaccine, Nivolumab	This is a multisite, phase I/II clinical trial in children and young adults with newly-diagnosed high-grade glioma (HGG), diffuse midline glioma (DMG) and recurrent HGG/DMG, Medulloblastoma (MB), or ependymoma (EPN) to determine the safety, immunogenicity, and efficacy of a CMV-directed peptide vaccine plus checkpoint blockade.
NCT05544526	1	2023–08–15	2025–12–01	CAR T Cells to Target GD2 for DMG	Recruiting	Diffuse Midline Glioma, H3 K27M-Mutant	Child	GD2 CAR T cells	The study will evaluate the feasibility of generating the ATIMP, the safety and tolerability of the GD2CAR T-cell therapy and how effectively GD2CAR T-cells engraft, expand and persist following administration in patients with DMG.
NCT05096481	2	2024–07–18	2028–06–15	PEP-CMV Vaccine Targeting CMV Antigen to Treat Newly Diagnosed Pediatric HGG and DIPG and Recurrent Medulloblastoma	Recruiting	High Grade Glioma, Diffuse Intrinsic Pontine Glioma, Recurrent Medulloblastoma	Both Child (DIPG) and Adult CNS Tumor Patients	PEP-CMV, Temozolomide	This study will address the question of whether targeting CMV antigens with PEP-CMV can serve as a novel immunotherapeutic approach in pediatric patients with newly-diagnosed high-grade glioma (HGG) or diffuse intrinsic pontine glioma (DIPG) as well as recurrent medulloblastoma (MB).
NCT05835687	1	2023–04–27	2028–03–01	Loc3CAR: Locoregional Delivery of B7–H3-CAR T Cells for Pediatric Patients With Primary CNS Tumors	Recruiting	Atypical Teratoid/Rhabdoid Tumor, Diffuse Midline Glioma, H3 K27M-Mutant, Ependymoma, High Grade Glioma, Glioblastoma, Medulloblastoma	Both Child (DIPG) and Adult CNS Tumor Patients	B7–H3-CAR T cells	Loc3CAR is a Phase I clinical trial evaluating the use of autologous B7–H3-CAR T cells for participants 21 years old with primary CNS neoplasms.
NCT05478837	1	2023–07–20	2029–08–31	Genetically Modified Cells (KIND T Cells) for the Treatment of HLA-A*0201-Positive Patients With H3.3K27M-Mutated Glioma	Recruiting	Diffuse Midline Glioma, H3 K27M-Mutant	Both Child (DIPG) and Adult CNS Tumor Patients	Autologous Anti-H3.3K27M TCR-expressing T-cells	This phase I, first-in-human trial tests the safety, side effects, and best dose of genetically modified cells called KIND T cells after lymphodepletion (a short dose of chemotherapy) in treating patients who are HLA-A\*0201-positive and have H3.3K27M-mutated diffuse midline glioma.
NCT04978727	1	2022–07–01	2027–04–30	A Pilot Study of SurVaxM in Children Progressive or Relapsed Medulloblastoma, High Grade Glioma, Ependymoma and Newly Diagnosed Diffuse Intrinsic Pontine Glioma	Recruiting	Medulloblastoma, Glioblastoma Multiforme, Anaplastic Astrocytoma, Anaplastic Oligodendroglioma, Anaplastic Ependymoma, Ependymoma, Diffuse Intrinsic Pontine Glioma	Both Child (DIPG) and Adult CNS Tumor Patients	SurVaxM	The primary objective of this trial is to assess the toxicity profile of SurVaxM in emulsion with Montanide plus sargramostim in children with relapsed or progressive medulloblastoma and high-grade glioma, ependymoma and non-recurrent diffuse intrinsic pontine glioma post-radiation therapy.
NCT06221553	1	2024–03–01	2026–03–01	Safety and Efficacy of Loco-regional B7H3 IL-7Ra CAR T Cell in DIPG	Recruiting	DIPG	Both Child (DIPG) and Adult CNS Tumor Patients	B7H3 specific CAR T cell with IL-7Ra signaling domain	A Phase 1 clinical trial to evaluate the safety and early efficacy of CAR T-cell with IL-7Ra signal targeting B7H3 in children with diffuse intrinsic pontine glioma (DIPG) patients after complete standard treatments.
NCT05717712	1	2023–01–04	2025–01–04	Oncolytic Virus Ad-TD-nsIL12 for Primary Pediatric Diffuse Intrinsic Pontine Glioma	Recruiting	DIPG	Both Child (DIPG) and Adult CNS Tumor Patients	Ad-TD-nsIL12	This is a drug safety assessment clinical trial with a 3 + 3 dose escalation design, to observe the safety, tolerability and toxicity of a novel oncolytic virus Ad-TD-nsIL12 intratumoral injection in primary DIPG patients (NCI-CTCAE V5.0).
NCT05717699	1	2023–01–04	2025–01–04	Oncolytic Virus Ad-TD-nsIL12 for Progressive Pediatric Diffuse Intrinsic Pontine Glioma	Recruiting	DIPG	Both Child (DIPG) and Adult CNS Tumor Patients	Ad-TD-nsIL12	This is a single-arm, single-center, drug safety assessment clinical trial with a 3 + 3 dose escalation design, to observe the safety, tolerability and toxicity of a novel oncolytic virus Ad-TD-nsIL12 intratumoral injection in progressive DIPG patients (NCI-CTCAE V5.0).
NCT04099797	1	2020–02–03	2027–02–01	C7R-GD2.CAR T Cells for Patients with GD2-expressing Brain Tumors (GAIL-B)	Recruiting	Diffuse Intrinsic Pontine Glioma, high grade glioma, medulloblastoma	Both Child (DIPG) and Adult CNS Tumor Patients	C7R-GD2.CART cells	The purpose of this study is to combine infusions into the vein in the first treatment cycle with infusions directly into the cerebrospinal fluid to find the largest safe dose of GD2–C7R T cells.
NCT06396481	1	2024–04–30	2026–12–30	Clinical Study of Allogeneic Vγ9 Vδ2T Cells in the Treatment of Brain Malignant Glioma	Not yet recruiting	DIPG, glioblastoma, medulloblastoma, ependymoma	Both Child (DIPG) and Adult CNS Tumor Patients	Vγ9 Vδ2T T cell	The main purpose of this study is to evaluate the safety and feasibility of allogeneic Vγ9 Vδ2 T cell therapy.
NCT04837547	1	2021–09–20	2025–09–01	PEACH TRIAL- Precision Medicine and Adoptive Cellular Therapy	Active, not recruiting	Diffuse Intrinsic Pontine Glioma, recurrent neuroblastoma	Child	Tumor-specific ex vivo expanded autologous lymphocyte transfer (TTRNA-xALT)	A Phase I open-label, multicenter study, to evaluate the safety, feasibility, and maximum tolerated dose of treating children with newly diagnosed DIPG or recurrent neuroblastoma with molecular targeted therapy in combination with adoptive cell therapy, Tumor-specific ex vivo expanded autologous lymphocyte transfer and Autologous G-CSF mobilized Hematopoietic Stem Cells (HSCs).
NCT04758533	1,2	2021–04–19	2026–04–01	Clinical Trial to Assess the Safety and Efficacy of AloCELYVIR With Newly Diagnosed Diffuse Intrinsic Pontine Glioma (DIPG) in Combination With Radiotherapy or Medulloblastoma in Monotherapy	Active, note recruiting	Diffuse Intrinsic Pontine Glioma, medulloblastoma	Child	AloCELYVIR	The aim of this study is to assess the safety and efficacy of AloCELYVIR, which consist in bone marrow-derived allogenic mesenchymal stem cells infected with an oncolytic Adenovirus, ICOVIR-5
NCT04254419	1	2025–03–04	2030–12–31	Phase 1 Study of Locoregional Injections of Ex Vivo Expanded Natural Killer Cells	Recruiting	High Grade Glioma	Both Child (DIPG) and Adult CNS Tumor Patients	NK cells	This study will investigate safety of TGFβi NK cell infusions.
NCT07031765	1	2025–07–01	2030–12–01	Peds CHAMP1ON - Hematopoietic Stem Cell And Monoclonal Antibody PD-1 Blockade for RecurreNt Pediatric High-Grade Glioma	Not yet recruiting	Recurrent High-grade Glioma, Grade IV Astrocytoma	Both Child (DIPG) and Adult CNS Tumor Patients	exHSC, Nivolumab	This study will investigate safety of ex vivo expanded CD34+ hematopoietic stem cells (exHSCs) administration.
NCT04323046	1	2020–10–02	2026–03–01	Immunotherapy Before and After Surgery for Treatment of Recurrent or Progressive High Grade Glioma in Children and Young Adults	Recruiting	Malignant Glioma, Recurrent Malignant Glioma	Both Child (DIPG) and Adult CNS Tumor Patients	Nivolumab	This phase I trial studies the side effects of nivolumab before and after surgery in treating children and young adults with high grade glioma that has come back (recurrent) or is increasing in scope or severity (progressive).
NCT06687681	2	2024–11–25	2025–11–25	Injection of Active Allogeneic Natural Killer Cells in Patients With Gliomas	Recruiting	High Grade Glioma (III or IV)	Both Child (DIPG) and Adult CNS Tumor Patients	NK cell therapy	This trial will study injection of active allogeneic NK cells.
NCT05660408	1,2	2025–03–12	2033–10–01	RNA Lipid Particles Targeting Pediatric Recurrent Intracranial Malignancies and Other systEmic Solid Tumors	Active, not recruiting	Recurrent High-grade Glioma	Both Child (DIPG) and Adult CNS Tumor Patients	pp65 RNA LP (DP1), pp65/tumor mRNA RNA-LP (DP2)	In this study, we will investigate the manufacturing feasibility, safety and immunologic activity of RNA-LP vaccine in patients with recurrent pulmonary or unresectable osteosarcoma and recurrent pHGG.
NCT06521567	1,2	2025–03–06	2029–10–05	A Study of Cobolimab Plus Dostarlimab in Pediatric and Young Adult Participants With Cancer	Recruiting	Diffuse Intrinsic Pontine Glioma (DIPG)	Both Child (DIPG) and Adult CNS Tumor Patients	Cobolimab, Dostarlimab	The goal of this interventional study is to determine the strength of cobolimab and dostarlimab that is most tolerated in children and young adults who have advanced solid tumors.
NCT06914479	1	2025–08–01	2030–08–01	Virus-Based Gene Therapy (AdV-HSV1-TK and AdV-Flt3L) in Combination With Valacyclovir for the Treatment of Pediatric and Young Adult Patients With Resectable, Recurrent Primary Malignant Brain Tumors	Not yet recruiting	Recurrent Diffuse Hemispheric Glioma, H3 G34-Mutant	Both Child (DIPG) and Adult CNS Tumor Patients	Ad-hCMV-Flt3L, Ad-hCMV-TK	This phase I trial tests the safety, side effects and best dose of AdV-HSV1-TK and AdV-Flt3L in combination with valacyclovir for the treatment of patients with primary cancerous (malignant) brain tumors that can be removed by surgery (resectable) and that have come back after a period of improvement (recurrent).

**Table 2:** An updated list of targeted antigens for the treatment of DMGs and their respective therapeutic approaches

Antigen	Antigen Type	Therapeutic Approach	Stage of Development	Study
H3.3K27M_26–35_	Histone antigen	Peptide vaccine	Clinical (Phase I/II)	Mueller et al., 2020 [19]
		TCR transduced T cells	Pre-clinical (murine model)	Chheda et al., 2018 [20]
B7–H3	Cell surface antigen	Monoclonal antibody	Clinical (Phase I)	Souweidane et al., 2018 [21]
		CAR T cell	Clinical (Phase I)	Vitanza at al, 2025 [22]
		Radioactive isotope and monoclonal antibody conjugate	Clinical (Phase I)	Souweidane et al., 2025 [23]
CD47	Cell surface antigen	Monoclonal antibody	Pre-clinical (murine model)	Gholamin et al., 2017 [24]
TIM-3	Cell surface antigen	Monoclonal antibody	Pre-clinical (murine model)	Ausejo-Mauleon et al., 2023 [25]
PD-1	Cell surface antigen	Monoclonal antibody	Clinical (Phase I/II)	Dunkel et al., 2023 [26]
CLTA-4	Cell surface antigen	Monoclonal antibody		
IDO	Intracellular enzyme	Small molecule	Clinical (Phase I)	Johnson et al., 2024 [5]
Multiple	Multiple	Oncolytic viral therapy	Clinical (Phase I)	Gállego Pérez‑Larraya et al., 2022 [27]
Multiple	Multiple	Autologous dendritic cells	Clinical (Phase I)	Benitez-Ribas et al., 2018 [28]
H3K27M_14–40_	Histone antigen	Peptide vaccine	Pre-clinical (murine model)	Ochs et al., 2017 [29]
			Clinical (Phase I)	Grassl et al., 2023 [30]
GD2	Cell surface antigen	CAR T cell	Clinical (Phase I)	Majzner et al., 2022 [31]
BCAN, EphA2, IL13Ra2	Cell surface antigens	CAR T cell	Pre-clinical (murine model)	Lakshmanachetty et al., 2024 [32]

## Tumor mutational burden & neoantigens

One of the key hurdles in advancing immunotherapy for DMG is the low tumor mutational burden (TMB). Similar to GBM, DMG are low-TMB, immune cold tumors that are poorly responsive to checkpoint blockade. Although GBM shows modestly enhanced immune infiltration, exhausted T cells, and chemokine-rich, PD-L1+ myeloid that promotes immunosuppression, DMG has profound lymphocyte deficiency and a resting myeloid microenvironment with extremely minimal inflammatory signaling. It’s often intact BBB also handicaps leukocyte and pharmacological access [33–35].

**Fig. 2:**
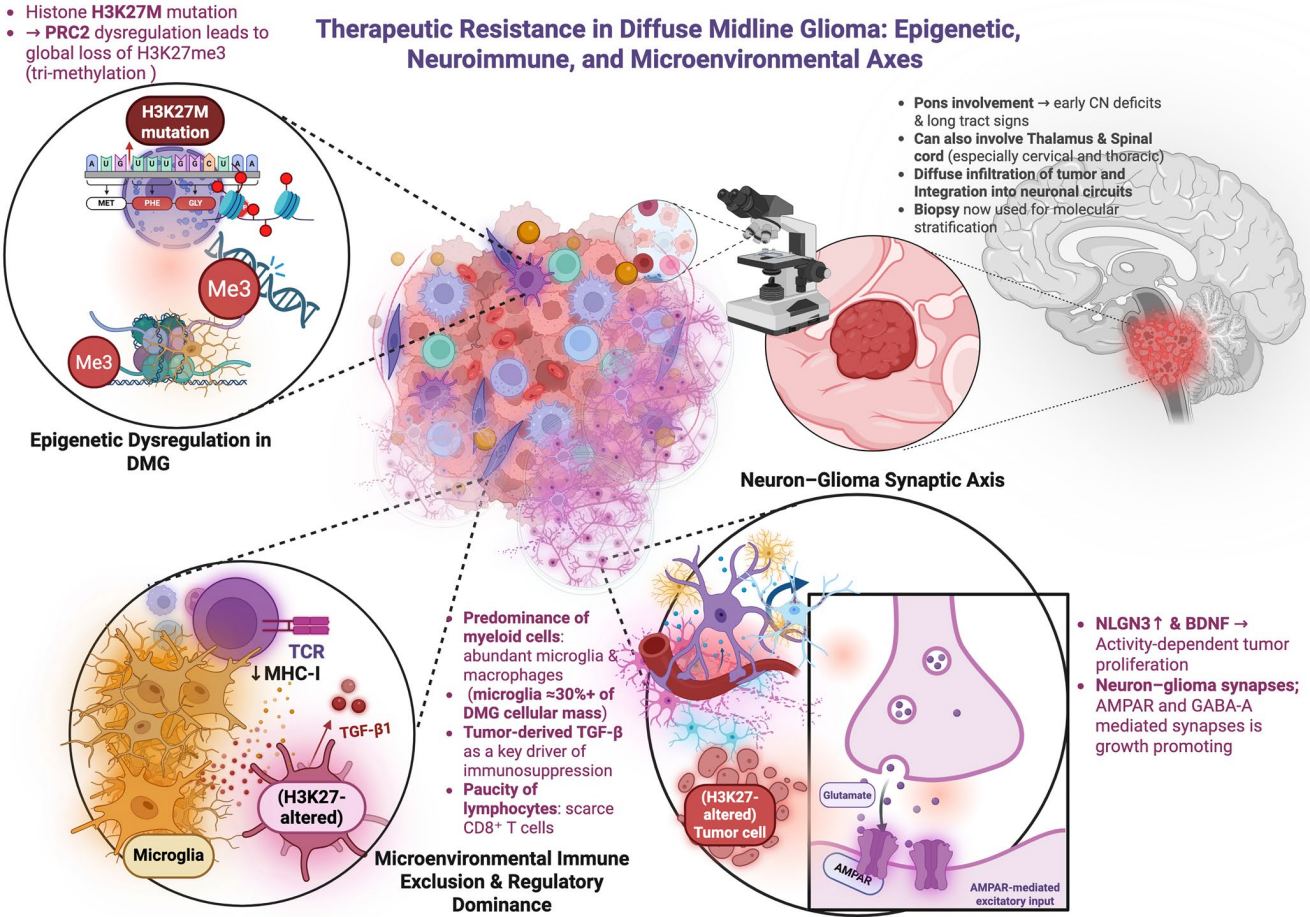
Integrated mechanisms of therapeutic resistance in diffuse midline glioma. This figure integrates the epigenetic, neuro-immune, and microenvironmental networks that sustain therapeutic resistance in diffuse midline glioma (DMG). DMG, most commonly caused by H3K27M histone mutations, is defined by pan-epigenetic dysregulation because of suppression of PRC2 complex and global loss of H3K27me3, redirecting transcriptional networks towards a malignancy. Midline structures develop tumors most predominantly at the pons but also thalamus, and spinal cord, in which the tumors expand diffusely and are integrated into neuronal networks. Neuron–glioma synaptic interactions, mediated through AMPA and GABA-A receptor–mediated transmission and regulated by activity-regulated molecules such as neuroligin-3 (NLGN3) and brain-derived neurotrophic factor (BDNF), underlie tumor expansion. The tumor immune microenvironment is characterised by a predominance of myeloid-lineage cells (microglia and monocyte-derived macrophages), largely devoid of cytotoxic lymphocytes, and rich in TGF-β1 secreted by the tumor. This environment, induced by low MHC-I expression and Treg function, generates an immunologically “cold” tumor milieu that limits efficacious anti-tumor immunity. Such immune, synaptic, and epigenetic components act in concert to enhance therapy resistance and disease progression in DMG. Figure created using biorender, accessed July 25th, 2025

Furthermore, The median TMB in DMG is typically < 1 mutation/Mb, limiting the number of potential neoantigens available for immune recognition [17, 34–36]. This low TMB correlates with poor immunogenicity and may contribute to the observed resistance to immune checkpoint blockade and other T cell-based therapies. Nonetheless, some mutations in DMG, such as the H3K27M alteration itself, offer attractive immunotherapeutic targets. Several preclinical and early-phase clinical studies have demonstrated the immunogenicity of H3K27M peptides, capable of eliciting both CD4+ and CD8+ T cell responses in vitro and in vivo [34, 35]. Neoantigens derived from recurrent driver mutations in ACVR1 and PPM1D may also be exploitable in select subsets [36]. Fig. 2 recapitulates mechanisms of therapeutic resistance in DMG.

Efforts are ongoing to leverage next-generation sequencing and mass spectrometry to identify low-abundance neoepitopes, post-translationally modified peptides, and tumor-specific splice variants in DMG [37, 38]. In addition, antigen presentation of the H3.3K27M mutation generates an immunogenic, HLA-A*02:01-restricted neoepitope that can be specifically targeted by engineered TCR-transduced CD8^+^ T cells, thus serving as an alternative immunogenic target even in the setting of low TMB [39]. These findings support the pursuit of cell-based and immunotherapy strategies such as personalized neoantigen vaccines and engineered T cell therapies tailored to the unique mutational and epigenetic signature of individual tumors (Table 2).

## Immune microenvironment & evasion

The immune microenvironment of DMG is considered “cold,” characterized by low levels of infiltrating lymphocytes, a paucity of antigen-presenting cells, and the presence of immunosuppressive myeloid populations [40, 41]. Single-cell RNA sequencing and spatial transcriptomics studies have confirmed the scarcity of tumor-infiltrating CD8+ T cells in DMG compared to adult glioblastoma, as well as the relative dominance of microglia- and monocyte-derived macrophages with tolerogenic phenotypes [42, 43].

The anatomical location of DMG within the brainstem imposes additional immunological constraints, particularly via the blood-brain barrier (BBB), which impedes the systemic delivery of therapeutic agents to the site of the tumor, and imposes a physical filter for peripheral immune invasion to the CNS [44].

Moreover, Zhu and colleagues demonstrate that the majority of patient-derived DMG tumor samples exhibit downregulation of MHC class I and class II molecules, further impairing T cell recognition and priming [45]. Epigenetic silencing of antigen presentation machinery and defects in interferon signaling contribute to this immune invisibility [46].

Tumor cell–expressed immunosuppressive ligands further reinforce local immune evasion. B7–H3 (CD276), a checkpoint molecule overexpressed in DMG, is associated with poor prognosis and reduced T cell activity [47]. Given these immune barriers, combinatorial strategies intended to target and alter the immunosuppressive microenvironment of DMG may be employed to sensitize the tumor to immune-based interventions.These include myeloid modulatory techniques which may shift tumor associated myeloid populations away from immunosuppressive phenotypes, or potentiate local inflammation and antigen release [48].

## Cancer neuroscience

In recent years, the emerging field of cancer neuroscience has uncovered a surprising axis between neurons and glioma. One seminal study by Venkatesh et al. (2015) found that increasing excitatory neuronal activity via optogenetic stimulation promoted greatly accelerated growth of pediatric glioma xenografts in mice [49]. This effect was mediated in part by paracrine growth factor signaling, including brain-derived neurotrophic factor (BDNF) and a shed form of the synaptic adhesion molecule neuroligin-3 (NLGN3) [49, 50] NLGN3 was found to be secreted in an activity-regulated manner and to stimulate oncogenic PI3K-mTOR signaling in glioma cells [49, 50]. Follow-up work found that patient-derived gliomas (including DIPG) fail to grow or exhibit delayed growth in NLGN3-knockout mice, highlighting their dependency on neuron-derived NLGN3 [50]. Importantly, inhibition of the metalloproteinase ADAM10, which cleaves and releases NLGN3, in these models inhibited tumor growth in-vivo.

The unexpected dependency of DMG and other high-grade gliomas on NLGN3 in the tumor microenvironment prompted a deeper exploration into the effects of NLGN3 on glioma cell states. This line of inquiry revealed that NLGN3 regulates the expression of numerous genes involved in synapses [50], sparking the hypothesis that the effects of neuronal activity on DIPG/DMG growth and progression may be mediated not only by paracrine factor signaling, but also by synaptic communication [49–51]. Electron microscopy studies identified clear synaptic structures forming between neurons and DIPG/DMG cells, and whole cell patch clamp electrophysiology revealed bona fide, electrophysiology functional synapses between neurons and DIPG/DMG cells, mediated by both AMPA receptors [51] and by GABA_A_ receptors [52]. These neuron-to-DMG synapses are growth-promoting [51–53], and exhibit hijacked mechanisms of synaptic plasticity [53].

Notably, recent evidence links glioma-neuron interactions to local immunosuppression. In glioblastoma, a high-grade glioma, regions with dense neuron-glioma synapses and that were demonstrated to be more functionally connected to the rest of the brain by intraoperative electrophysiology studies [54] were found to be enriched with immunosuppressive TAMs and lacked antigen-presenting function [55]. A causal relationship between the high functional connectivity between brain and tumor and the immune-suppressed environment remains to be demonstrated, but this association adds to important questions about how neuron-immune cell-cancer cross-talk may contribute to an immune-suppressed tumor microenvironment [56].

## Immune Checkpoint Inhibitors (ICIs)

Immune checkpoint inhibitors (ICIs), targeting CTLA-4, PD-1, and PD-L1, have revolutionized the treatment of several adult malignancies but have shown limited efficacy in DMG, due in part to their low levels of expression in the tumor-immune microenvironment [40, 45, 57]. Interestingly, one preclinical study reported that systemically administered anti-TIM-3 antibodies penetrated the tumor site in murine models of DIPG/DMG, attributed to extensive neovascularization that increased vascular permeability [58]. However, it is important to note that the models used HSJ-DIPG-007 xenografts, which require immunodeficient hosts and PDGF-B-driven syngeneic models, either lack an intact immune system or exhibit a disrupted BBB. This contrasts with DMG, which often retains an intact BBB as demonstrated by contrast-enhanced MRI using gadolinium-based contrast agents [34]. The more pronounced efficacy of anti-TIM-3 therapy in these models, therefore, likely may reflect their leaky vasculature rather than true antibody penetrance across an intact BBB. As such, early clinical trials of ICIs in DMG have been largely disappointing, with no significant survival benefit observed in monotherapy settings. For instance, a phase I trial of nivolumab in children with recurrent CNS tumors, including DIPG, demonstrated acceptable safety but minimal clinical activity [59].

The limited efficacy of ICIs in DMG may be attributed to multiple factors: low TMB, sparse T cell infiltration, lack of preexisting immune priming, and an immunosuppressive tumor microenvironment. Furthermore, analysis of pre- and post-irradiated DIPG autopsy samples lacks upregulated neoantigen expression and T cell infiltration, indicating that radiation, which canonically activates the immune response in cancer therapy, may not demonstrate the same effect in DIPG [40]. In contrast to adult glioblastomas, PD-L1 expression in DMG is relatively low and heterogeneous, further challenging the rationale for single-agent checkpoint blockade [42]. To overcome these limitations, current approaches focus on combination therapies. Trials such as PNOC022 (NCT05009992) and NCT04049669are investigating the efficacy of ICIs or small inhibitory molecules in combination with radiation or epigenetic agents in newly diagnosed DMG [60, 61].

Other strategies aim to prime the immune response via oncolytic viruses, vaccines, or dendritic cell activation prior to ICI administration [40, 48, 62]. Targeted inhibition of immunosuppressive pathways such as CSF1R or IDO may further enhance checkpoint sensitivity [63, 64]. Collectively, these insights underscore the need for continued development of combinatorial strategies that not only enhance checkpoint sensitivity but also effectively circumvent the intact blood–brain barrier of DMG to enable adequate drug delivery.

## Vaccine-based immunotherapies

Vaccination strategies in DMG focus on the clonal H3K27M mutation as a tumor-specific neoantigen. Short peptide vaccines (~10 amino acids) encompassing the H3K27M epitope have been shown to induce mutant-specific CD8^+^ T cells in HLA-A*02:01^+^ patients [19]. In one pilot study, an H3.3K27M_26–35_ short peptide vaccine elicited circulating CD8^+^ T cells in HLA-A2^+^ patients aged 3–21 with DMG [19]. However, because short peptides directly load onto MHC class I molecules, their efficacy is limited by HLA subtype restriction, most notably HLA-A*02:01, and recent evidence suggests that these epitopes may not be endogenously presented by DMG tumor cells, raising concerns about whether the induced T cells can effectively recognize and kill tumor cells [65]. In contrast, long peptides (~20–30 amino acids) must be taken up and processed by antigen-presenting cells, leading to broad MHC class II presentation and robust CD4^+^ T-helper responses. A 27-mer H3K27M peptide vaccine spanning amino acids 14–40 demonstrated strong CD4^+^ T cell immunogenicity across multiple HLA types in humanized models [29]. This vaccine, when given repeatedly to adult DMG patients (often with PD-1 checkpoint blockade), was safe and induced mutation-specific T cell responses in 5/8 patients, including one case of sustained complete remission beyond 31 months [30].

Dendritic cell (DC) vaccines, which work by priming T cells against loaded tumor antigens or patient-specific neoantigen peptides, have also been of interest. In one ongoing phase I/II trial (NCT04911621), Wilms Tumor 1 (WT1) oncoprotein mRNA-loaded autologous monocyte-derived DC vaccines are being evaluated for the treatment of HGG and DIPG. However, DIPG is “immunologically cold” tumors with low baseline T cell infiltration and cytokine signaling, potentially limiting vaccine efficacy [40]. Despite these limitations, vaccine-based immunotherapies are a potentially valuable avenue of exploration, and several current clinical trials, as mentioned earlier, are evaluating new mechanisms to combat these shortcomings.

## Car T-cell therapies

Chimeric antigen receptor (CAR) T cells, a form of adoptive cellular therapy, have been at the forefront of emergent therapeutics for CNS maligancnies over the recent years [66]. There are currently numerous DMG immunotherapy research with multiple CAR T targets under active investigation. GD2-specific CAR T cells were the first tested in patients, given the high GD2 expression on H3K27M^+^ DMG cells and complete tumor responses observed in preclinical models [67]. In a landmark phase I study (NCT04196413), eleven patients with diffuse midline gliomas received GD2-CAR T cells intravenously, with subsequent intracerebroventricular administration for those patients who demonstrated benefit. Of these eleven patients, nine demonstrated clinical benefit, including marked improvement in neurological deficits, and 4 of the eleven patients demonstrated a greater than 50% reduction in tumor volume. One patient exhibited a complete and durable response [68]. At the time of data cutoff, median overall survival was 17.9 months for pontine DMG patients and 20.9 months for all DMG patients [68], but please note that it is difficult to compare overall survival data in a patient cohort with strict eligibility criteria to unselected historical cohorts. Inducing therapeutic inflammation in critical structures such as the brainstem, or spinal cord is not without risk, and all patients exhibited variable degrees of transient, localized neurological symptoms attributable to inflammation at the site of the tumor termed “tumor inflammation-associated neurotoxicity (TIAN)” [31, 68, 69]. Patients developed concurrent cytokine release syndrome following IV, but not ICV, administration of GD2-CAR T cells, and CRS represented the dose-limiting toxicity for IV administration of GD2-CAR T cells for DIPG/DMG.

Other CAR T targets are being explored in parallel. B7–H3 (CD276) CAR T cells have shown particular promise for pediatric CNS tumors, including DMGs which uniformly express B7–H3 at high levels. In the BrainChild-03 phase 1 clinical trial, 21 children with DIPG, a subset of DMG, received over 250 intracerebroventricular (ICV) repetitive B7–H3 CAR T cell infusions via intraventricular catheter [22]. The median overall survival from CAR T initiation was 10.7 months. Although several patients in this cohort remain alive beyond 3–4 years on maintenance dosing, only one of these long-term survivors had a confirmed DMG/DIPG diagnosis, while others had either unbiopsied tumors or IDH1-mutant high-grade gliomas, which carry a more favorable prognosis. Therefore, conclusions regarding the long-term survival benefit of B7–H3 CAR T cell therapy in confirmed DMG/DIPG patients should be interpreted with caution. To address tumor heterogeneity and antigen escape, novel CAR T designs are moving beyond single targets, such as a “Quad-CAR” T cell product co-targeting B7–H3, EGFR806, HER2, and IL13Rα2 which is in development (NCT05768880). Dual CAR approaches and logic-gated CAR T cells are also being engineered to require two tumor antigens for activation, increasing specificity and reducing the risk of antigen-negative relapse. For example, one synNotch CAR T construct, which utilizes a “priming” antigen followed by a “killing” antigen to prevent off-target toxicity, named α-BCAN synNotch α-EphA2/IL-13 Rα2 CAR (B-SYNC) T cells efficiently targeted DMG/DIPG cells in vitro [32].

However, these constructs are still in the early stages of development and have not been clinically validated in DMG and DIPG patients.

## Other adoptive cellular approaches (NK cells, TILs, viral therapies)

In conjunction with CAR T-cell therapy, adoptive treatments involving natural killer (NK) cells and tumor-infiltrating lymphocytes (TILs) are investigated in DMG and other high-grade pediatric gliomas. NK cell strategies utilize MHC-independent killing and are susceptible to TGF-β–mediated inhibition, a feature of the DMG microenvironment [70]. Two current trials are paradigmatic of this trend: a Phase I trial of locoregional TGFβ-inhibited NK cells infused intraventricularly or intratumorally through Ommaya or VP shunt (NCT04254419) and a Phase II trial of intrathecal infusion of allogeneic NK cells in high-grade gliomas, including DMG (NCT06687681). Combined, they evaluate the safety, dosing, and immune-phenotypic persistence of NK products in the CNS compartment [71, 72]. Conversely, TIL-based treatments have significant translational challenges in DMG, the inferior tumor mutational burden, low levels of endogenous T-cell infiltration, and unfavorable resectability for cell collection, parameters reduce feasibility compared to extracranial solid tumors. New strategies, including epigenetic or oncolytic preconditioning and local delivery, could potentially augment lymphocyte homing and antigenicity. According to our initially established research criteria and paper scope, modalities themselves are now placed briefly in context here, and corresponding trials are cited in Table 1.

Furthermore, New viral platforms are gaining traction in diffuse midline glioma (DMG) as direct tumor lysis and immunostimulatory vehicles. Oncolytic adenovirus DNX-2401 proved to be viable and reprogrammed the immune-microenvironment in a phase 1 DIPG trial in The New England Journal of Medicine (NCT03178032). Coadjutant strategies involve IL-12-expressing adenoviral vectors (Ad-TD-nsIL12) in pediatric DIPG early-phase trials (NCT05717712, NCT05717699), and an IL-12–encoding, engineered HSV-1 (M032) under investigation for de novo DMG (NCT07076498). These gene-transduced viral modalities have the capacity to combine direct oncolysis with local cytokine delivery, possibly reconstituting DMGs immunologically “cold” microenvironment into a more inflamed, therapeutically targeted condition [20, 21, 23]. While viral therapeutics is a fast-moving area, a comprehensive review of every trial in viro- and immunotherapy is beyond the scope of this manuscript; each modality, CAR T, NK, TIL, and viral, would deserve a separate review on its own. Here, we instead give a brief synthesis that integrates representative strategies across modalities to bring attention to convergent mechanisms of immune activation in DMG.

## Radioimmunotherapy

Radiation therapy, the use of external beams of radiation to target cancer cells, is a significant cornerstone in the treatment of malignancies and has been an integral component of the Stupp protocol since 2005 [24]. Radioimmunotherapy (RIT), instead, utilizes radioactive isotopes conjugated to monoclonal antibodies as a method to deliver radiation directly to cancer cells, minimizing off-target toxicity [25]. For the treatment of DMG, B7–H3 (CD276) has been a promising target for reasons stated above in the adoptive cellular therapies section. In a phase I trial of RIT, children with leptomeningeal or metastatic CNS tumors received multiple infusions of ^131^I-omburtamab, a murine anti–B7–H3 monoclonal antibody. The treatment was well tolerated, as acute toxicities were mostly mild (headache, nausea, fever) and grade 3/4 thrombocytopenia was the most common hematologic effect. Importantly, patients treated for CNS neuroblastoma (NB) leptomeningeal disease had a significantly prolonged median PFS of ~7.5 years, significantly longer than historical controls of ~13.1 months [26].

For DMG/DIPG, one alternative strategy has been direct injection of RIT into the tumor via convection-enhanced delivery (CED). Souweidane et al. reported a phase I dose-escalation study of ^124^I-omburtamab, delivered via MR-guided CED into the pons of 50 pediatric DIPG patients shortly after radiotherapy [28]. The approach was generally safe with no grade 4/5 CNS toxicities, although several dose-limiting events, namely grade 3 edema, occurred. Importantly, patients in this group had a median overall survival of 15.3 months from diagnosis, surpassing the typical survival of around 11 months and pointing to a possible therapeutic advantage. The ratio of radiation concentrated in the tumor compared to the rest of the body was exceptionally high, demonstrating that convection-enhanced delivery enabled highly focused treatment. These early investigations show that targeting B7–H3 with radioimmunotherapy is feasible in DMG. Ongoing research aims to optimize treatment schedules and dosages, as well as to assess combination strategies, such as adding radiosensitizers or immunotherapies, for improved outcomes. While other molecular targets like GD2 and HER2 are under evaluation in laboratory models, B7–H3 continues to be the most promising candidate for RIT in DMG.

## Towards a systems-level paradigm: integrative immunoepigenetic and neuroimmune targeting

The trend of DMG research has more and more emphasized that a reductionist, monotherapy-oriented approach is not enough to address the multi-level pathobiology of such tumors. As our overall understanding of DMG biology develops, an improved conceptual model, one that positions the tumor within its dynamic epigenetic, immunologic, and neurophysiologic context, is needed. The intersection of H3K27M-induced dysregulation of chromatin, growth-promoting neuron-glioma interactions, and immune exclusion defines a uniquely recalcitrant tumor in which each of these axes of therapeutic resistance reenforces the others. Single-agent treatment against any one dimension, epigenetic, immune, or neuronal, has predictably delivered only minor and transitory gains, as demonstrated in many early-phase trials [5, 19, 22, 28–32, 39, 58, 59, 73–75]. The future involves the implementation of rational, combinatorial strategies for disrupting the mutually reinforcing dependencies between these axes. In particular, their co-administration with epigenetic modulators (such as HDAC or EZH2 inhibitors) to restore circumstantially partial antigen presentation and immunogenicity [76–79], with immunotherapies that span checkpoint blockade to regionally delivered CAR T cells, is an attractive method to bypass the immune inertia of the DMG microenvironment [80]. Similarly, the rising knowledge that crosstalk between neurons and gliomas actively drives DMG growth and invasion, with possible further influences on the suppressive tumor immune microenvironemnt leads to the prediction that therapeutic interventions designed to interfere with neuron-glioma and neuron-glioma-immune interactions would be of double utility: incapacitating tumor growth while rendering the microenvironment less hospitable for immune evasion (Table 3). The development of these regimens, though, will require not only intense preclinical modeling but also exact manipulation of the timing, sequence, and regional delivery of agents to prevent toxicity and capitalize on windows of weakness in the TME [81].

**Table 3:** Proposed multimodal therapeutic strategies targeting key axes of resistance in DMGs

Axis of Resistance	Mechanism	Therapeutic Target(s)	Examples of Agents/Strategies	Rationale for Combination Therapy
Epigenetic Dysregulation	H3K27M-mediated loss of H3K27me3, immune evasion, stemness	EZH2, HDAC, PRC2, BET proteins	Tazemetostat (EZH2i), Panobinostat (HDACi), BET inhibitors	Epigenetic reprogramming can restore MHC expression and tumor immunogenicity
Immune Exclusion	Immune desert or suppressive myeloid microenvironment	Immune checkpoints, myeloid regulators, CAR T cells	Anti-PD-1/PD-L1, Anti-GD2 CAR T, IDO inhibitors, CSF1R inhibitors	Epigenetic priming + immunotherapy can synergize to overcome immune inertia
Neuron–Glioma Interactions	Activity-dependent growth, synaptogenesis, AMPA signaling	NLGN3, TSP1, AMPAR pathways	NLGN3 inhibitors, AMPAR antagonists	Disrupts glioma proliferation and reverses immune suppression linked to neuronal signaling
Tumor Microenvironment & Delivery Barriers	Dense stroma, BBB, hypoxia	Direct delivery, BBB disruption	Convection-enhanced delivery, focused ultrasound, intrathecal CAR T	Improves local drug concentration and reduces systemic toxicity

Equally important is the development of adaptive therapeutic strategies predicated on longitudinal, noninvasive monitoring. The promising performance of plasma- and cerebrospinal fluid-based liquid biopsies in the identification of H3K27M mutant DNA [82–85] and new radiogenomic methods of monitoring immune infiltration and epigenetic status in situ [45] points toward a future where therapy is dynamically adjusted to real-time biological signals instead of fixed upfront planning. One can envision a clinical trial architecture in which induction with cancer neuroscience or epigenetic agents, those which target tumor–neuron interactions, such as glutamatergic signaling inhibitors or neuronal activity-modifying drugs, “prepare” tumor and TME, as evidenced by enhanced MHC presentation and immune cell infiltration, followed by optimally delivered cellular immunotherapies (Table 4) [78, 86]. These adaptive regimens would require the identification and field validation of strong surrogate biomarkers of response and resistance, and greater insight into how systemic and local immune dynamics evolve under treatment pressure. More broadly, these findings necessitate a conceptual transition beyond the think of DMGs as simple chemoresistant, surgically occult tumors, to one of systems-level disease state that is epigenetically plastic, neuroimmune-embroiled, and susceptible to potentially integrated multimodal disturbance. Future progress will be more likely to depend on less any one new agent than our ability to devise a biologically logical, temporally and spatially optimal therapeutic program, a transition as much intellectual as technical.

**Table 4:** Key emerging tools for adaptive, systems-level management of DMGs

Tool/Approach	Application in DMG Management	Advantages	Current Limitations/Considerations
Liquid Biopsies (cfDNA, CSF-tDNA)	Real-time monitoring of H3K27M mutations, clonal evolution, response to therapy	Minimally invasive, longitudinal, dynamic	Sensitivity still variable, needs standardization
Radiogenomics & Advanced MRI	Correlate imaging with immune infiltration, epigenetic states	Non-invasive, spatially resolved	Needs validation of predictive value
Epigenetic Priming Followed by Immunotherapy	Sequential induction of immunogenicity, then immune attack	Rational synergy, targets tumor plasticity	Optimal timing/sequencing yet to be defined
Theranostic Delivery Platforms	Trackable targeted therapies with concurrent imaging	Confirms delivery, enables adaptive adjustments	Infrastructure-intensive, early-phase evidence
Integrated Multimodal Trial Designs	Adaptive protocols based on biomarkers & longitudinal monitoring	Personalized, efficient	Requires regulatory and logistical innovation

## Conclusion

DMGs remain among the most therapeutically intractable pediatric malignancies due to their infiltrative growth patterns, “cold” tumor microenvironments, and anatomical restraints preventing surgical resection. Nonetheless, recent advancements in immunotherapeutic platforms, ranging from neoantigen-targeted peptide vaccines and intraventricular CAR T cell regimens to B7–H3 directed radioimmunoconjugates, have demonstrated early signs of biological and, in select cases, clinical activity. Furthermore, multidisciplinary insights from cancer neuroscience have further implicated neuron-glioma synaptogenesis as a contributor to local immunosuppression, highlighting novel axes for clinical therapies in the future. Recent advancements in immunological therapies, as summarized in this review, highlight several promising avenues through which immunotherapies can be applied to DMGs. Ongoing translational efforts that incorporate these principles into rationally designed clinical trials will be critical to advancing durable and meaningful responses in this otherwise uniformly fatal disease. As more patients begin receiving CNS-delivered immunotherapies, long-term follow-up becomes critical, not only to assess survival, but also to monitor for late immune, endocrine, and neurocognitive sequelae [87]. To discover clinically significant immunotherapies for the treatment of DMGs, robust experimental models and long-term monitoring must become standard components of therapeutic development.

## Data Availability

No datasets were generated or analysed during the current study.
